# Indents

**Published:** 1923-06

**Authors:** 


### INDENTS

**☞Brief Articles of Technical or Scientific Value will receive notice and comment. Contributions invited.**

THE ENDOCRINE GLANDS. In the course of an article summarizing recent advances in knowledge of the functions of the ductless glands, Dr. Albert Crosby, in the *Dental Cosmos,* gives the following account of the effects of the internal secretions of these glands upon the teeth and jaws:

"Hypofunction (of the parathyroids) may produce tetany. In tetany the hair, nails, skin and enamel are trophically disturbed. There is hypoplasia of the enamel, causing the formation of horizontally transverse ridges. The incisor teeth show opaque spots on their anterior surfaces. Regular teeth imply balance between the anterior and posterior pituitary. Teeth crowded together, with high arches, means relatively more posterior than anterior pituitary. A good firm lower jaw suggests the gland that produces acromegaly, as does wide spacing of the teeth.

"Absent, abnormal or small lateral incisors indicate abnormal or poorly developed gonads and internal genitalia.

"In anomalies of the thymus, and of the thyroid and parathyroids, because of the calcium disturbances, there may be lateral erosions of the enamel.

"The color, shape and size of the teeth is thought to be determined by the predominance of one gland or another. Thymus insufficiency is the cause of the crescent-shaped notch. This occurs in 'white' teeth, and should not be confused with the notched teeth of specific origin.

"Thyroid, finely active, produces teeth of pearly whiteness, regular arrangement. Adrenal teeth are yellowish. A predominance of this secretion produces an aggressive character and large canines."—*The Dental Surgeon.*

---

CLEANING THE TEETH. There are few subjects upon which there is so much divergence of opinion than this. There exist, of course, extremists on both sides. Some authorities would rely entirely upon a correct form of diet, and would discard such things as toothbrushes altogether; others, on the other hand, care little about the diet provided that the mouth and teeth are properly cleaned after each meal.

We do not belong to either of these camps. We regard toothbrushes as very useful implements, but we would not rely entirely upon them.

It is, of course, more or less obvious that if the mouth and teeth be properly cleaned after every meal, and no food be allowed to remain in contact with the teeth for more than a few moments, the nature of the food can not matter very much. But the question at once arises as to whether it is always possible to perform this cleaning operation perfectly, and let us say at once that we do not think it is.

First of all there are those whose business arrangements do not permit of such operations being performed, at any rate after the mid-day meal. But apart from such considerations we do not believe it to be possible, by the use of a toothbrush, to render the mouth and teeth completely free from collections of food after a meal, unless due consideration is paid to the type of food consumed, and the order in which it is consumed. Thus the fundamental principles so ably set forward by Dr. Sim Wallace and others, require to be acted upon intelligently by the man in the street, if his teeth are to remain reasonably free from disease.

Provided that the diet is chosen with due regard to these principles, then the toothbrush may fulfil a very useful function. There are, however, certain risks connected with the instrument which should be fully realized by those who employ it. Unless adequate precautions are taken to sterilize it, the toothbrush may be a fertile source of infection. Sterilization by boiling is, of course, the most efficient method, but this shortens the life of the average toothbrush very considerably. Paraform tablets are extremely useful in this connection. These tablets give off formalin very slowly; if the toothbrush be kept in a case together with one or two paraform tablets, the formalin evolved will suffice to destroy any organisms likely to be present on the brush. This is a simple method, which anyone can use without difficulty. It is to be hoped that the importance of sterilizing the toothbrush may become more widely recognized. A clean brush is a valuable prophylactic instrument, whereas a dirty one is a very dangerous weapon.—*The Dental Surgeon.*

